# Sulfatases, in Particular Sulf1, Are Important for the Integrity of the Glomerular Filtration Barrier in Zebrafish

**DOI:** 10.1155/2019/4508048

**Published:** 2019-07-22

**Authors:** Heiko Schenk, Anna Masseli, Patricia Schroder, Patricia Bolaños-Palmieri, Michaela Beese, Jan Hegermann, Jan Hinrich Bräsen, Hermann Haller

**Affiliations:** ^1^Department of Nephrology and Hypertension, Hannover Medical School, Hannover, Germany; ^2^Mount Desert Island Biological Lab, Bar Harbor, ME, USA; ^3^Department of Nephrology and Hypertension, University of Erlangen-Nurnberg, Erlangen, Germany; ^4^Research Core Unit Electron Microscopy, Hannover Medical School, Hannover, Germany; ^5^Institute of Pathology, Nephropathology Unit, Hannover Medical School, Hannover, Germany

## Abstract

The 6-O-endosulfatases (sulfs) are important enzymatic components involved in the regulation of heparan sulfate by altering the sulfatation pattern. Specifically in the kidney, sulfs have been implicated in the glomerular podocyte-endothelial cell crosstalk and in the preservation of the glomerular filtration barrier (GFB) in different mouse models. Since it has been shown that in zebrafish larvae, Sulf1, Sulf2a, and Sulf2b are expressed in the pronephric kidney we set out to establish if a reduction in sulf expression leads to GFB dysfunction. Here, we show that a reduced sulf expression following morpholino (MO) induced knockdown in zebrafish larvae promotes damage to the GFB leading to renal plasma protein loss from the circulation. Moreover, a combined knockdown of Sulf1, Sulf2a, and Sulf2b is associated with severe morphologic changes including narrowing of the fenestration between glomerular endothelial cells as well as thickening of the glomerular basement membrane and podocyte foot process effacement, suggesting that glomerular damage is an underlying cause of the circulatory protein loss observed after MO injection. Additionally, we show that a decrease in sulf expression reduces the bioavailability of VegfA in the glomerulus of the pronephros, which may contribute to the structural changes observed in the glomeruli of morphant fish. Furthermore, consistent with previous results, knockdown of the sulfs is associated with arteriovenous malformations in particular in the tail region of the larvae. Overall, taken together our results suggest that 6-O-endosulfatases are important in the preservation of GFB integrity and a reduction in their expression levels induces phenotypic changes that are indicative of renal protein loss.

## 1. Introduction

The 6-O-endosulfatase enzymes (sulfs) are key regulators of heparan sulfate (HS) exerting their modulating function through the modification of the sulfatation pattern by selectively removing 6-O-sulfate of HS predominantly in the highly sulfated domains [1–4]. The interaction between HS and vascular endothelial growth factor (VEGF) is mediated directly through the sulfs on the basis of the degree of 6-O-sulfatation on HS polysaccharide chains [5–9]. The regulatory function of the sulfs on growth factor signaling including VEGFA-associated pathways is mediated directly in the extracellular matrix (ECM) or at the cell surface [9]. In a previous study using HUVECs, siRNA induced knockdown of* hSULF1* abrogated arterial marker expression in response to VEGFA stimulation [9]. Furthermore, zebrafish larvae with a reduced sulf1 mRNA expression had arterial malformations including arteriovenous shunts [9]. Given that these malformations were prevented by coinjection of ectopic* vegfa165* mRNA, these findings support the hypothesis that Sulf1 expression is necessary for Vegf mediated angiogenesis [9].

Analogous to mouse glomeruli which express both Sulf1 and Sulf2 [1, 3], zebrafish express three homologs of the sulfs: Sulf1, Sulf2a, and Sulf2b, in the pronephric ducts [10]. However, the sulf pronephros expression pattern appears to be dynamic since not all sulf homologs are present in parallel during pronephros development [10].

It has been established in mouse models that endothelial cell morphology and function are influenced by the paracrine mechanisms of podocytes, specifically signaling to the endothelium via the podocyte-derived VEGFA which binds to its receptors, namely, VEGFR1 (Flt1) and VEGFR2 (Flk1) [11–13]. Schumacher* et al.* demonstrated that VEGF signaling was reduced in Sulf1^−/−^; Sulf2^−/−^ mice, indicating that both sulfatases regulate the VEGF-mediated crosstalk between podocytes and endothelial cells [13].

In a model of diabetic kidney disease using streptozotocin (STZ) in mice, typical characteristics of diabetic nephropathy including mesangial matrix accumulation and foot process effacement were present in Sulf1^−/−^; Sulf2^−/−^ mice; however, WT mice having received the same STZ-treatment displayed neither mesangial matrix accumulation nor podocyte injury [1]. Takashima* et al.* attributed the mesangial hypercellularity and matrix expansion to the reduction of glomerular VEGF signaling in Sulf1^−/−^; Sulf2^−/−^ mice, since mesangial proliferation occurs after depletion of podocyte-derived VEGF [1, 11, 12].

To further evaluate the potential role of sulf1 in proteinuric kidney disease, we used the currently published zebrafish nephrosis model using puromycin aminonucleoside (PAN) via treatment in the fish water [14]. PAN, as previously published in a rat model, causes a specific toxic injury to podocytes resembling human minimal change disease or FSGS including foot process effacement, disruption of the actin cytoskeleton, and expression changes of slit diaphragm associated proteins [14, 15].

In this study, we demonstrate that sulfatases, in particular Sulf1, are pivotal players in maintaining the integrity of the glomerular filtration barrier in zebrafish. The lack of sulfatase expression reduces the glomerular presence of Vegfa in the zebrafish pronephros and the injection of Vegfa 165 zebrafish protein can partially prevent injury to the glomerular filtration barrier after Sulf1^KD^. Therefore, this study provides evidence for the relevance and potential mechanisms for the role of sulfatases, in particular Sulf1, in the regulation of glomerular integrity in zebrafish.

## 2. Materials and Methods

### 2.1. Morpholino and Protein Injection

Injections of sulf1 splice acceptor [9], sulf2a ATG blocking, sulf2b splice donor, and scrambled-control morpholinos (Gene Tools, Philomath, OR) or Vegfa 165 recombinant zebrafish protein (R&D Systems, Minneapolis, MN) into fertilized zebrafish eggs were carried out at the 1- to 2-cell stage, as previously described [16, 17]. MOs were ordered from Gene Tools (Philomath, OR) and were diluted 1:1 in injection buffer (20mmol/L HEPES, 200 mmol/L KCl, and 0.75% phenol red).

### 2.2. Electron Microscopy

Zebrafish larvae were fixed at 96 hpf in solution D and embedded in EPON (recipe/protocol from EMS, Hatfield, PA). Further preparation of the zebrafish embryos for TEM was carried out as stated in previously published protocols [18]. Imaging was done using a Morgagni TEM (FEI, Eindhoven, NL), operated at 80 kV.

### 2.3. Measurement of the Fluorescence Intensity in the Zebrafish Retinal Vessel Plexus

As a method to evaluate the integrity of the GFB, we measured a GFP-tagged vitamin D binding protein derived from* Tg(l-fabp:eGFP-DBP)* zebrafish in the retinal vessel plexus of the zebrafish larvae at 96 hpf, as previously described [16]. The maximum fluorescence intensity of gray-scale images of the retinal vessels was measured. The subsequent analysis was carried out with ImageJ (Version 1.48v, National Institutes of Health, Bethesda, MD) and reported in arbitrary units (AU).

### 2.4. PAN Model

Zebrafish eggs were collected within 30 min of spawning followed by injection with the respective morpholino or the scrambled controls as described above. Prior to treatment, the chorions were removed from the embryos manually with forceps. As described previously [14], at 46 hpf, zebrafish were treated with either 2 mg/ml or 8 mg/ml PAN, which was added into the fish medium with 1% DMSO. Until 96 hpf, zebrafish were kept in the PAN containing medium followed by the fluorescence intensity measurement in the retinal vessel plexus. DMSO added to the fish medium in the appropriate concentration served as control.

### 2.5. qPCR of Zebrafish Larvae

Purification of total RNAs from zebrafish larvae was done with RNeasy Plus Mini Kit (QIAGEN, Venlo, Netherlands) according to the manufacture's protocol. Reverse transcription of 1*μ*g RNA was done using Oligo(dT)primer (Promega, Madison, WI, USA), and Random primers (Promega, Madison, WI, USA) that were incubated at 70°C for 10 min followed by retrotranscription in M-MLV RT buffer (Promega, Madison, WI, USA), with dNTPs (Roche, Mannheim, Germany), and M-MLV reverse transcriptase (Promega, Madison, WI, USA) at 42°C for 90 min and at 70°C for 10 min. Sybr green-based qPCR was performed using the TaKaRa SYBR Premix Ex Taq™ II.

### 2.6. Zebrafish Immunofluorescence Staining on Paraffin Sections

Zebrafish larvae were fixed in 2% paraformaldehyde (PFA), dehydrated in ascending concentrations of ethanol (30%, 50%, 70%, 80%, 90%, and 100%) and transferred to 100% SUB-X (Leica Biosystems, Nussloch, Germany) for 4 hours before embedding in paraffin for 4 hours. After embedding in paraffin, 4 *μ*m sections were cut on a rotational microtome (Leica RM 2245). The sections were deparaffinized in Histo-Clear tissue clearing agent for 3 x 5 min and rehydrated in a descending ethanol series (100%, 96%, 70%, 50%, and finally A. dest.). For antigen retrieval, the slides were treated with trypsin (Merck KGaA, Darmstadt, Germany) at 37°C for 8 min. After blocking, 1:25 rabbit monoclonal anti-VEGFA (Abcam, Cambridge, UK) antibody was incubated for 1 h at room temperature. Donkey anti-Rabbit IgG (H+L) antibody, Alexa Fluor 488 (Thermo Fisher Scientific, MA, USA) was used as secondary antibody for 1 h at room temperature in a 1:500 dilution. Rhodamine Wheat Germ Agglutinin (WGA) (Vector Laboratories, CA, USA) in a 1:2000 dilution was also used. Nuclei staining was performed with DAPI present in the Immunoselect Antifading Mounting Medium (Dianova, Hamburg, Germany). Images were taken with a 10x and a 63x objective on a Leica DMI6000 B microscope (Leica Biosystems, Nussloch, Germany).

### 2.7. Dextran Microinjection and In Vivo Confocal Imaging of the Vasculature

Tricaine (Sigma-Aldrich, Munich, Germany) was used at a concentration of 0.1–0.5% (in E3 solution) to anesthetize fish prior to microinjection and confocal microscopy. 4.6 nl of the dextran (2000kDa) labeled with tetramethylrhodamine (Invitrogen, San Diego, CA) dissolved in PBS (5mg/ml) was injected into the cardinal vein. Following the dextran injection, confocal imaging of age and size matched embryos was carried out at 48 and 72 hpf. For this, the embryos were mounted and single color z series were acquired on an Olympus Fluoview FV1000 confocal laser scanning microscope system (Olympus, Tokyo, Japan).

### 2.8. Statistics

The statistical analysis of data was performed using GraphPad Prism 7 software. Error bars correspond to SEM. We used one way ANOVA followed by Tukey's multiple comparisons test or student's t test to compare the results of each single test group. A P value of ≤0.05 was considered statistically significant. *∗*p≤0.05, *∗∗*p≤0.01, *∗∗∗*p≤0.001, *∗∗∗∗*p≤0.0001.

### 2.9. Animal Ethics

All animal work was performed according to the NIH Guideline for the Care and Use of Laboratory Animals. The animal protocol was approved by the MDI Biological Laboratory IACUC #17-03 and is in line with the MDIBL international assurance # D16-00341.

## 3. Results 

### 3.1. Knockdown of Sulf1, Sulf2a, and Sulf2b Causes Arteriovenous Malformations

**Figure 1:**
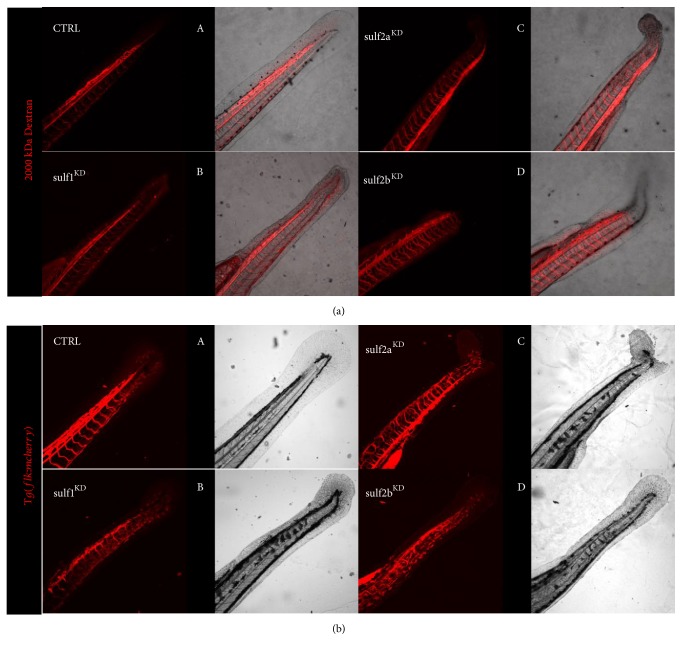
Sulfatases knockdown induces circulatory malformations. (a) Fluorescence images of a WT zebrafish larvae at 48 hpf after injection of tetramethylrhodamine labeled dextran (MW 2000kDa) in the cardinal vein. CTRL-MO injected larvae exhibit regular development of the tail circulation. Sulf1^KD^ larvae display minimal blood circulation in the tail and potential premature shunting between the caudal artery and the caudal vein. Sulf2a^KD^ larvae display minor disruptions of the tail vasculature. Sulf2b^KD^ larvae exhibit a minor injury to the tail vasculature including reduced circulation in the distal tail areas. (b) Fluorescence images of* Tg(flk1-mCherry)* larvae at 72 hpf. CTRL-MO injected larvae display regular development of the tail circulation. Sulf1^KD^ larvae display a discontinuation of the caudal artery in the tail vasculature indicative of an arteriovenous shunt between caudal artery and caudal vein. The blood circulation in the distal tail is diminished. Sulf2a^KD^ larvae exhibit disruptions of the tail vasculature and reduced blood circulation in the distal tail. Sulf2b^KD^ larvae exhibit signs of vascular injury in the tail including reduced circulation in the distal tail areas.

Circulatory defects as a result of reduced Sulf1 in zebrafish have been previously shown by Gorsi* et al*. [9]. Therefore in order to further characterize the vascular defects in larvae with reduced sulfatase expression, we performed a MO-induced knockdown of Sulf1, Sulf2a, and Sulf2b and evaluated vascular integrity following 2000 kDa dextran injection at 48 hpf in WT zebrafish or mcherry-flk expression at 72 hpf in* Tg(flk:mcherry)* larvae. Sulf1 morphant embryos exhibit impaired blood circulation in the tail, and an aberrant connection between caudal artery and caudal vein is present in the majority of Sulf^KD^ larvae at both 48 and 72 hpf (Figures 1(a)B and 1(b)B). Sulf2a morphant larvae display a more variable phenotype and show mild to more severe disruptions in the caudal artery but also in the intersegmental vasculature of the tail at both 48 and 72 hpf (Figures 1(a)C and 1(b)C). Sulf2b morphant larvae also exhibit an impaired vascular integrity in the tail regions showing a reduced blood circulation. Analog to the Sulf2a morphants, Sulf2b^KD^ induces damage to the intersegmental vasculature (Figures 1(a)D and 1(b)D). Scrambled morpholino injected CTRL larvae neither display an impairment of the venous or arterial blood vessels in the tail region at time point 48 nor 72 hpf (Figures 1(a)A and 1(b)A).

### 3.2. Knockdown of Sulf1, Sulf2a, and Sulf2b Leads to Circulatory Protein Loss, Pericardial Effusion, and Yolk Sac Edema Indicative of Renal Protein Loss

**Figure 2:**
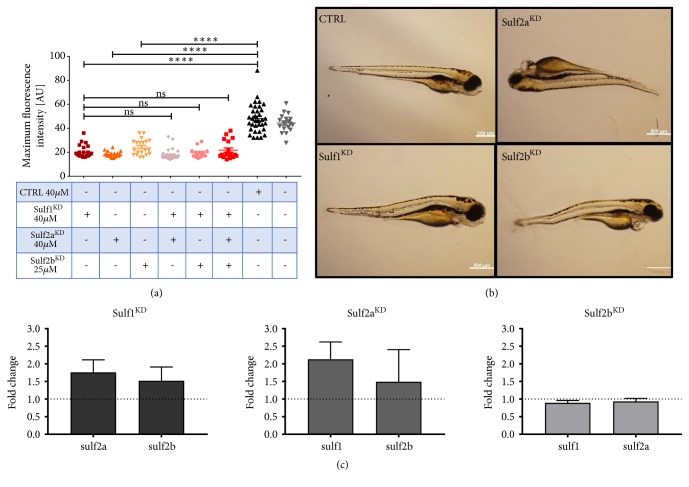
Morpholino induced knockdown of sulf1, sulf2a, and sulf2b leads to a reduction of circulating eGFP-DBP, pericardial effusion, and yolk sac edema. (a) Measurement of the maximum fluorescence intensity (AU) in the retinal vessel plexus at 96 hpf. Zebrafish embryos were injected in the yolk sac at the 1-2 cell stage with either a scrambled control (CTRL) or with sulf1 MO, sulf2a MO, or sulf2b MO. Additionally, a combined morpholino induced knockdown of Sulf1 and Sulf2a, Sulf1 and Sulf2b and Sulf1, Sulf2a and Sulf2b. In comparison with the CTRL larvae, Sulf1^KD^, Sulf2a^KD^, and Sulf2b^KD^ larvae display significant reduction of the maximum fluorescence indicating a loss of eGFP-DBP from the blood circulation. Neither combined Sulf1^KD^ and Sulf2a^KD^ or Sulf1^KD^ and Sulf2b^KD^ nor all of the three sulfatases were able to decrease the maximum fluorescence intensity below the maximum of a single knockdown of the respective sulfatases. n≥15 for all groups; each circle shows the maximum fluorescence for one individual fish; error bars are SEM; nonsignificant (ns), *∗∗∗∗*p≤0.0001; ANOVA and Tukey's multiple comparison test. (b) Phenotype analysis of the CTRL shows no sign of pericardial effusion, yolk sac edema, or curvature of the tail. Knockdown of Sulf1, Sulf2a, and Sulf2b each lead to marked pericardial effusion, moderate to severe yolk sac edema, and mild curvature of the tail. Scale bar = 500 *μ*m. (c) Real-time polymerase chain reaction quantification (qPCR) of mRNA levels of the sulfs after MO induced knockdown; mRNA levels are depicted as a fold change relative to the control-MO injection. mRNA expression of the sulfs is normalized to the housekeeping gene* hprt*. Sulf1^KD^ induces higher* sulf2a* and* sulf2b* expression and Sulf2a^KD^ leads to an increase of* sulf2a* mRNA while* sulf2b* expression is highly variable. Sulf2b^KD^ does not lead to a compensatory* sulf1* or* sulf2a* mRNA expression. Error bars represent the SEM between three independent experiments (n = 3).

MO-induced downregulation of either Sulf1, Sulf2a, or Sulf2b causes a significant decrease in the maximum fluorescence intensity of circulating eGFP-DBP in the retinal vessel plexus of* Tg(l-fabp:eGFP-DBP)* larvae at 96 hpf (Figure 2(a)). A combined knockdown of the sulfs however did not further reduce the maximum fluorescence intensity in the retinal vessel plexus (Figure 2(a)). The scrambled MO injected zebrafish neither displayed a reduction of the fluorescence in comparison with noninjected zebrafish nor did they show phenotypic traits indicative of renal protein loss including pericardial effusion or yolk sac edema (Figures 2(a) and 2(b)). On the contrary, sulf1^KD^, sulf2a^KD^, and Sulf2b^KD^ larvae exhibit marked pericardial effusion and moderate yolk sac edema in addition to a mild curvature of the tail at 96 hpf (Figure 2(b)). In order to determine if there is a compensatory effect of sulf expression following MO-knockdown, morphant larvae were collected at 96 hpf and mRNA levels were assessed by qPCR. Both Sulf2a and Sulf2b mRNA show an increase after sulf1^KD^ (Figure 2(c)); and Sulf1 expression is also elevated following Sulf2a^KD^; however it seems that this upregulation is not sufficient to impede the development of a renal phenotype. Additionally, Sulf2b^KD^ did not lead to a compensatory increase of either Sulf1 or Sulf2a (Figure 2(c)).

### 3.3. Combined Knockdown of Sulf1, Sulf2a, and Sulf2b Using TEM Reveals Loss of Glomerular Endothelial Fenestration, Thickening of the GBM, and Podocyte Foot Process Effacement

**Figure 3:**
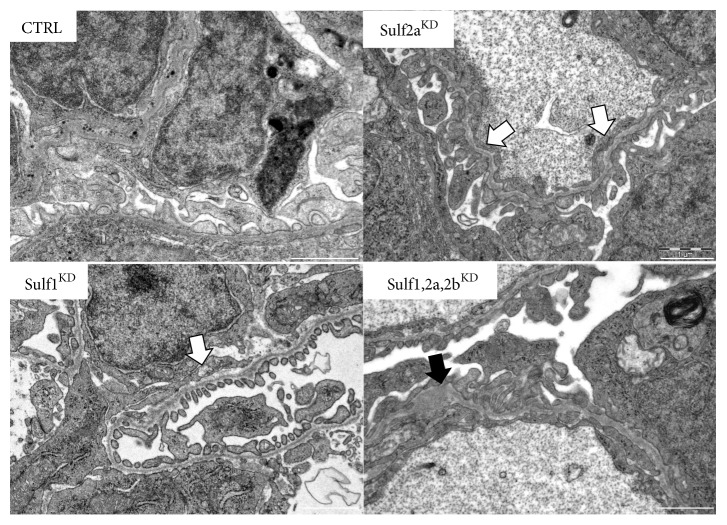
Ultrastructural impairment of the glomerular filtration barrier in sulf^KD^ larvae. Transmission electron microscopy imaging was used to detect ultrastructural changes in glomeruli after Sulf1^KD^, Sulf2a^KD^, and Sulf2b^KD^ induced by MO injection in* Tg(l-fabp:eGFP-DBP)* transgene zebrafish embryos at a one to two cell stage. Collection of larvae for imaging was done at 96 hpf. CTRL zebrafish injected with a control morpholino (CTRL-MO) show no sign of glomerular injury. Sulf1^KD^ larvae display an intermittent reduction of glomerular endothelial fenestration and partial podocyte foot process effacement. Sulf2a^KD^ causes only mild podocyte foot process effacement but decreases the fenestration of the endothelium markedly. A striking glomerular phenotype is detected in larvae with a combined Sulf1, 2a and 2b^KD^. Larvae display an irregular widening of the glomerular basement membrane while the endothelial fenestration is distinctly diminished. Effaced foot processes can furthermore be detected in the combined knockdown larvae. Black arrow: widening of the glomerular basement membrane, white arrow: reduced endothelial fenestration. Scale bar = 1 *μ*m.

Ultrastructural analysis of the larval glomerulus by TEM showed that scrambled MO injection in* Tg(l-fabp:eGFP-DBP)* transgene zebrafish (CTRL) did not induce injury to the glomerular filtration barrier (Figure 3). However, Sulf1^KD^ caused a partial reduction of the glomerular endothelial fenestration in addition to focal podocyte foot process effacement. The knockdown of Sulf2a showed similar effects since partial foot process effacement was present. The impact of the Sulf2a^KD^ on the endothelium was more distinct, as shown by a marked reduction in the fenestration of the endothelial cells (Figure 3). However, the most striking signs of glomerular function impairment were detected in the Sulf1, Sulf2a, and Sulf2b combined knockdown larvae. Focal thickening of the GBM was visible and fenestration of the endothelium was strongly diminished while the foot processes of the podocytes appeared focally effaced (Figure 3). These phenotypic changes indicate that a reduction in the expression of all three sulfs further impairs the morphology of the glomerulus when compared to a single sulf^KD^.

### 3.4. Knockdown of Sulf1, Sulf2a, and Sulf2b Leads to Loss of Vegfa Protein Expression in the Larval Zebrafish Glomerulus

**Figure 4:**
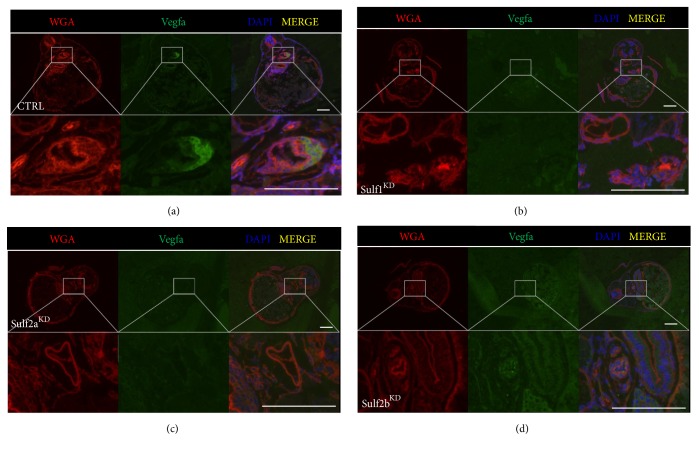
Morpholino induced knockdown of sulf1, sulf2a, and sulf2b leads to reduced Vegfa expression in the glomerulus at 96 hpf. Zebrafish embryos were injected in the yolk sac at the 1-2 cell stage with either a scrambled control (CTRL) or sulf1 MO, sulf2a MO, or sulf2b MO. Fluorescence images of zebrafish larvae paraffin sections detecting WGA (red) and Vegfa (green) expression show Vegfa protein expression in the glomerulus of the pronephros in CTRL larvae. In Sulf1^KD^ and Sulf2a^KD^ larvae, no expression of Vegfa is detectable in the glomerulus. Sulf2b^KD^ larvae show a reduced Vegfa expression in the pronephric glomerulus. Scale bar = 100 *μ*m.

Previous work by Gorsi* et al.* showed that Sulf1 regulates the differentiation of the vasculature in zebrafish in a Vegfa dependent manner [9]. Since the reduction of sulfatase expression causes circulatory protein loss (Figure 2(a)), we wanted to examine whether Vegfa expression in the glomerular region of the pronephros is affected at 96 hpf after MO-induced Sulf1^KD^, Sulf2a^KD^, and Sulf2b^KD^. In order to assess this, we detected the presence of Vegfa in zebrafish glomeruli after MO-knockdown; analysis of the anatomical structures was carried out by costaining with the lectin WGA which aids in the detection of membranous glycoproteins. The glomeruli of control-MO injected larvae show a strong Vegfa protein expression (Figure 4(a)). In contrast, however, knockdown of Sulf1, Sulf2a, and Sulf2b all shows a striking reduction on Vegfa expression in the larval glomerulus (Figures 4(b)–4(d)). Overall these data show that Vegfa expression in the zebrafish glomerulus is dependent on the presence of the sulfatases.

### 3.5. Knockdown of Sulf1 Induced Circulatory Protein Loss Can Be Partially Prevented by Coinjection of Vegfa 165 Recombinant Protein

**Figure 5:**
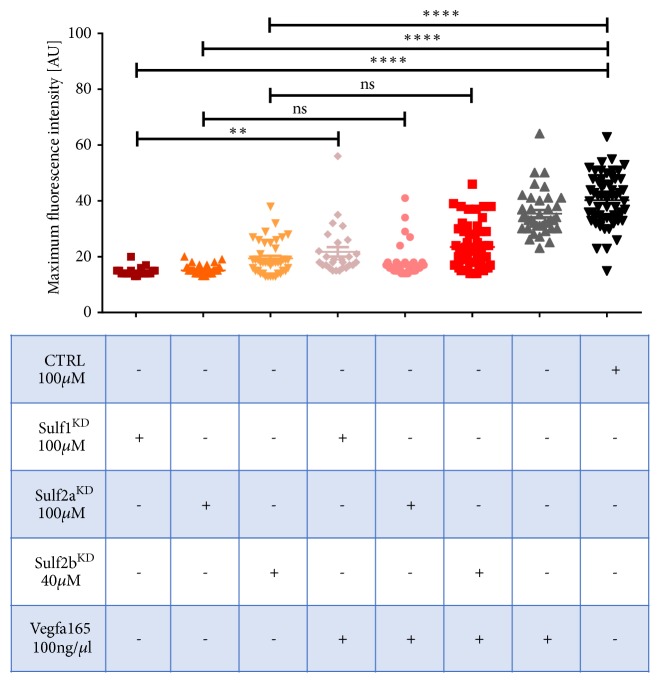
High dose but not low dose Vegfa 165 recombinant protein partially prevents eGFP-DBP loss from circulation in Sulf1^KD^ larvae but not in Sulf2a^KD^ and Sulf2b^KD^ larvae. Measurement of the maximum fluorescence intensity (AU) in the retinal vessel plexus at 96 hpf. Zebrafish embryos were injected in the yolk sac at the 1-2 cell stage with either a scrambled control (CTRL) or with sulf1 MO, sulf2a MO, or sulf2b MO and coinjected with Vegfa 165 protein. MO-induced Sulf1^KD^, Sulf2a^KD^, and Sulf2b^KD^ induced maximum fluorescence intensity reduction indicative of eGFP-DBP loss, coinjection of Vefga 165 protein 100 ng/*μ*l partially prevented the fusion protein loss in Sulf1^KD^ larvae. The Vefga 165 protein 100 ng/*μ*l coinjection in Sulf2a^KD^ and Sulf2b^KD^ larvae did not prevent eGFP-DBP loss.

Given that Gorsi* et al.* were able to show that Sulf1^KD^ induces arteriovenous malformations in zebrafish larvae which were prevented by the coinjection of Vegfa165 and that Sulf1^−/−^; Sulf2^−/−^ mice have reduced VEGF signaling [1, 9, 13], we aimed to determine whether ectopic Vegfa 165 could restore the leaky GFB in* Tg(l-fabp:eGFP-DBP)* larvae with reduced sulfatase expression. As stated above, diminished glomerular Vegfa expression was detected in Sulf1^KD^, Sulf2a^KD^, and Sulf2b^KD^ (Figure 4). Additionally, induced knockdown of the sulfatases caused a reduction in the eGFP-DBP fusion protein detected in the retinal vessel plexus, which was partially prevented in Sulf1^KD^ zebrafish using 100 ng/*μ*l of Vegfa 165 recombinant zebrafish protein (Figure 5), while 1 ng/*μ*l did not prevent fusion protein loss (Figure S1). In Sulf2a^KD^ and Sulf2b^KD^ larvae, the coinjection of Vegfa 165 did marginally increase the amount of circulating fluorescent protein; however this did not reach a statistical significance. This indicates that solely restoring the Vegfa presence by ectopic supplementation is not sufficient to correct the circulatory protein loss (Figures 5 and S1). Vegfa 165 injection alone did not induce a reduction of eGFP-DBP neither at 100 nor at 1ng/*μ*l (Figures 5 and S1).

### 3.6. Coinjection of Vegfa165 Recombinant Zebrafish Protein Partially Prevents Yolk Sac Edema and Pericardial Effusion in Sulf1 Morphants But Not in Sulf2a and Sulf2b Morphants

**Figure 6:**
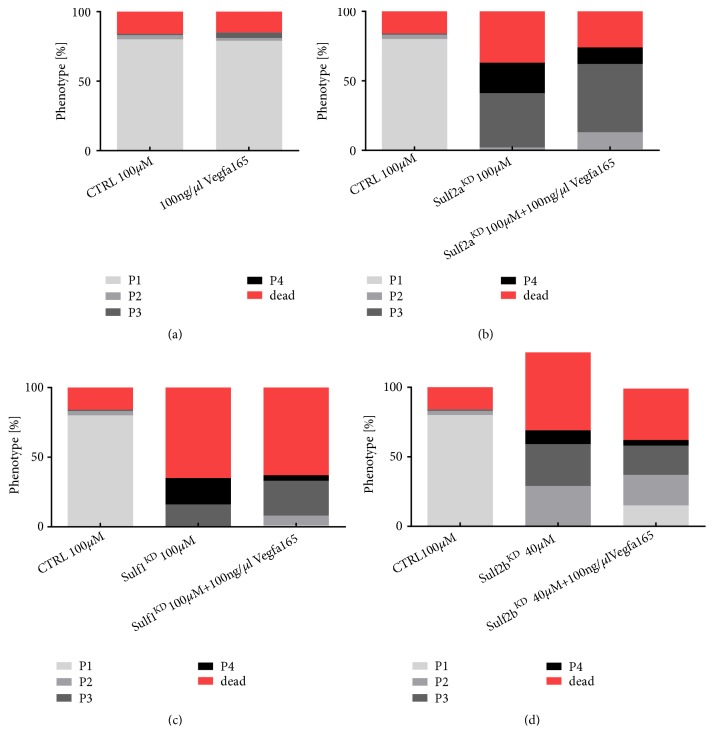
Knockdown of the sulfatases induces severe edema in the majority of larvae and decreases survival of larvae at 96 hpf. Zebrafish embryos were injected in the yolk sac at the 1-2 cell stage with either a scrambled control (CTRL) or sulf1 MO, sulf2a MO, or sulf2b MO. Data shown as the percentage of zebrafish in each of the phenotypic categories according to edema severity (ranging from P1=no edema, to P4=very severe edema) and death. (a) In comparison with CTRL larvae, Vegfa 165 injected zebrafish show no difference in mortality and presence of edema. (b) Sulf1^KD^ larvae display a percentage of dead fish of 65% compared to 63% in sulf1^KD^ Vegfa 165 coinjected larvae. The percentage of severe edema (P4) is reduced from 19% to 4% in the Vegfa 165 coinjected larvae. (c) Sulf2a^KD^ zebrafish have a 37% fatality rate as opposed to a 26% rate of Vegfa 165 coinjected larvae. Furthermore, the percentage of severe edema (P4) is reduced from 22% in Sulf2a^KD^ larvae to 12% in the Vegfa 165 coinjected fish. (d) In contrast to the Sulf1^KD^ and Sulf2a^KD^, Sulf2b^KD^ larvae display a lower fatality rate with 32% in comparison to the Vegfa 165 coinjected larvae with 37%. However, the coinjection of Vegfa 165 reduces the amount of P4 and P3 zebrafish and leads to a normal phenotype (P1) in 15% while none of the Sulf2b^KD^ fish display a normal phenotype.

To determine phenotype changes indicative of renal protein loss, we evaluated each larva at 96 hpf regarding pericardial effusion and yolk sac edema. Depending on the severity of edema and pericardial effusion, a normal appearance was scored as a P1 while most severe edema and pericardial effusion was scored as P4. Furthermore, the amount of dead fish at 96 hpf was determined. Analog to the evaluation of the eGFP-DBP in the retinal vessel plexus, ectopic Vegfa 165 expression was associated neither with edema or pericardial effusion nor with a higher mortality at 96 hpf in comparison with scrambled MO (CTRL) injected* Tg(l-fabp:eGFP-DBP) *fish (Figure 6(a)). The coinjection of Vegfa 165 in sulf1^KD^ zebrafish did not reduce death at 96 hpf but decreased the number of larvae displaying a severe edema P4 phenotype (Figure 6(b)). Since the coinjection of Vegfa 165 did not correct the phenotype to the degree of the CTRL, only a partial prevention of edema and pericardial effusion can be attributed to ectopic Vegfa administration (Figure 6(b)). A minor reduction in the death rate at 96 hpf and a reduction of P4 larvae was detected in larvae that were coinjected with Vegfa 165 (Figure 6(b)). Vegfa 165 ectopic supplementation in Sulf2a^KD^ fish led to a minor reduction in mortality and decreased the amount of P4 larvae at 96 hpf but did not correct the phenotype to a level comparable to the CTRL larvae (Figure 6(c)). In Sulf2b^KD^ larvae, the coinjection of Vegfa 165 did not reduce death but slightly lowered the amount of P4 and P3 larvae (Figure 6(d)). Overall, the ectopic expression of Vegfa 165 recombinant protein only showed a tendency to slightly improve the phenotype of all sulf morphant larvae.

### 3.7. Knockdown of Sulf1 Induces Additional Circulatory Protein Loss in the PAN Nephrosis Model

**Figure 7:**
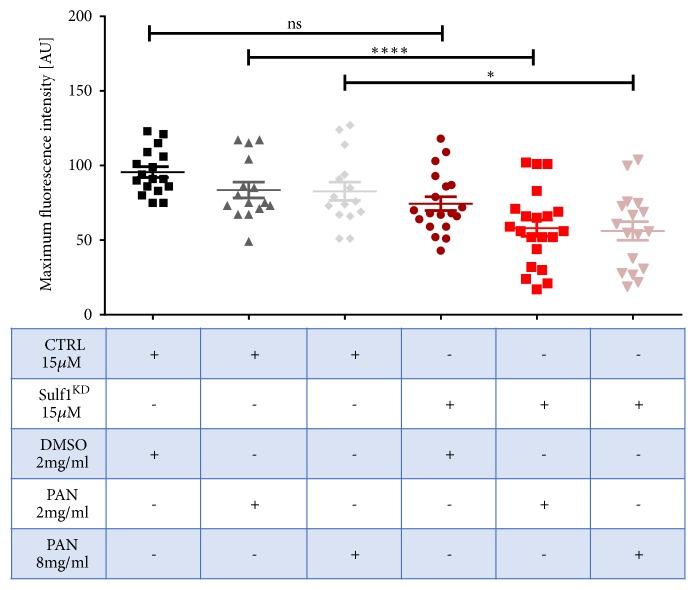
PAN treatment causes additional loss of eGFP-DBP in Sulf1^KD^ larvae suggesting an additive injury to the glomerular filtration barrier. Low dose sulf1 MO injection (15 *μ*M) in the yolk sac of 1-2 cell stage zebrafish with the respective DMSO control in the surrounding medium did not induce a significant reduction of the maximum fluorescence intensity compared to a scrambled control (CTRL) with DMSO control. The addition of 2 or 8 mg/ml of PAN in the surrounding medium however induced a reduction of the maximum fluorescence indicating additional fusion protein loss.

The PAN nephrosis model is an established model in rats and zebrafish [14, 15]. PAN itself damages podocytes morphologically resembling minimal change glomerulonephritis or FSGS [15]. To determine whether reduction of sulf1 expression could function as modulator to increase the susceptibility for an impaired GFB, we used the previously published PAN nephrosis model in zebrafish larvae [14]. Sulf1^KD^ with a reduced MO concentration (15 *μ*M) was not sufficient to reduce the maximum fluorescence intensity on its own, but PAN treated Sulf1^KD^* Tg(l-fabp:eGFP-DBP) *larvae at 96 hpf showed a reduced fluorescence intensity indicating circulatory eGFP-DBP fusion protein loss (Figure 7). This implies that a reduction in Sulf1 expression may contribute to the destabilization glomerular architecture making the podocytes more susceptible to injury, as previously suggested [1].

## 4. Discussion 

In this study we show that sulfs not only are of relevance for the development of the vasculature of zebrafish, but also play a pivotal role in the maintenance of the glomerular filtration barrier. Not only is Knockdown of Sulf1, Sulf2a, and Sulf2b causative of eGFP-DBP fusion protein loss of* Tg(l-fabp:eGFP-DBP)* larvae indicative of proteinuria, but also it may function as a second hit to the GFB in the PAN nephrosis model. However, since fusion protein loss cannot solely be attributed to leakage of the glomerular filtration barrier, and edema as well as pericardial effusion being rather unspecific, an ultrastructural analysis using transmission electron microcopy is necessary to determine if the protein loss occurs as a result of an impaired GFB. Electron micrographs of the glomeruli of a single sulf knockdown has shown moderate narrowing of the fenestrae between glomerular endothelial cells while mild phenotypic changes were present in the podocyte foot processes. In spite of not inducing further fusion protein loss, the sulf triple knockdown larvae compared to a single knockdown of the sulfs showed a more severe injury in particular to the endothelial cells and a more distinct thickening of the GBM in comparison with the single knockdown larvae. These phenotypic changes indicate that the fusion protein loss from the circulation occurs as a result of the impaired GFB. Furthermore, reduced Sulf1 and Sulf2a expression in zebrafish larvae was associated with a slight compensatory increase in mRNA expression of the other sulfs. These findings support the hypothesis that a combined sulf knockdown may promote a more severe glomerular impairment phenotype. Our results are consistent with previously reported ultrastructural analysis of murine kidneys which has shown that Sulf1^−/−^; Sulf2^−/−^ mice present with manifold indicators of glomerular disease including thickening of the GBM, an increase of the subendothelial space while fenestrae between endothelial cells are either narrow or completely lost, and podocyte foot processes appeared effaced [1, 13]. Since the ultrastructural phenotype of Sulf-deficient mice partly resembles the glomerular injury caused by impaired VEGF signaling in humans, other groups have also aimed to investigate the role played by VEGF signaling in Sulf1^−/−^; Sulf2^−/−^ mice [1, 13, 19, 20]. Here we show that in zebrafish, reduced sulf expression results in impaired Vegfa expression in the glomerulus which may be a contributing factor leading to proteinuria. Ectopic administration of Vegfa 165 also led to a partial prevention of the eGFP-DBP fusion protein loss in Sulf1^KD^ zebrafish larvae and showed a tendency to reduce protein loss in Sulf2b^KD^ larvae. However, given that podocytes produce high amounts and, therefore, are responsible for maintaining the bioavailability of Vegfa for endothelial cells, even unspecific damage to the podocyte itself may reduce Vegfa signaling to endothelial cells. As an example, it has been shown that endothelial cells have significantly fewer fenestrations as a result of podocyte cell ablation induced by the use of a nitroreductase (NTR)/metronidazole (MTZ) model [18, 21, 22]. As proposed by Schumacher* et al*., reduction of VEGF binding to heparan sulfate may induce paracrine VEGF signaling in the glomerular endothelium [13], while a decrease in VEGFA may be causative for the narrowing of the fenestrae of the endothelial cells as demonstrated in a murine VEGFA conditional knockout model [20]. In conclusion, our results support the concept that sulfs are highly relevant for maintaining the glomerular filtration barrier and mediate this function in a VegfA dependent manner. Furthermore, our data are the first to characterize the glomerular function of the sulfs adding more insight to glomerular endothelial cell crosstalk using a zebrafish model system.

## Data Availability

The datasets generated and analyzed during the current study are available from the corresponding author on reasonable request.

## Supplementary Materials

Supplementary Materials Figure S1: Measurement of the maximum fluorescence intensity (AU) in the retinal vessel plexus at 96 hpf. Zebrafish embryos were injected in the yolk sac at the 1-2 cell stage with either a scrambled control (CTRL) or with sulf1 MO, sulf2a MO, or sulf2b MO and coinjected with Vegfa 165 protein. MO-induced Sulf1^KD^, Sulf2a^KD^, and Sulf2b^KD^ induced maximum fluorescence intensity reduction indicative of eGFP-DBP loss; however, coinjection of Vegfa 165 1 ng/*μ*l did not prevent fusion protein loss. Primers: Primer sequences for zebrafish: hprt forward: 5'ACCAAACACTATGCGGCTG3' and reverse: 5'GTGTCCACCCATGTCCTTCA3'. sulf1 forward: 5'AACCCACGATACAGACCTCG3' and reverse: 5'CCCCCTCAAACTCGACTGAC3' sulf2a forward: 5'ATTGTGAACCCACAGCGTCT3' and reverse: 5'TTCAGTTTCTTCTTTGTGAGTGCT3' sulf2b forward: 5'GACACCATGAGGTGCCAGAG3' and reverse: 5'TAAAACTGTGACCCGCGACG3'. Morpholinos: sulf1: GTAGTCCTGGTAGTGGTAGAATAAT sulf2a: CCATGACCTTAACCAGTGCTCCACC sulf2b: TTCAGTCTAATTCACCTTCTCCACA CTRL scrambled: CCTCCTACCTCAGTTACAATTTATA.

## References

[1] Takashima Y., Keino-Masu K., Yashiro H. (2016). Heparan sulfate 6- *O* -endosulfatases, Sulf1 and Sulf2, regulate glomerular integrity by modulating growth factor signaling. *American Journal of Physiology-Renal Physiology*.

[2] Lamanna W. C., Frese M., Balleininger M., Dierks T. (2008). Sulf loss influences N-, 2-O-, and 6-O-sulfation of multiple heparan sulfate proteoglycans and modulates fibroblast growth factor signaling. *The Journal of Biological Chemistry*.

[3] Nagamine S., Tamba M., Ishimine H. (2012). Organ-specific sulfation patterns of heparan sulfate generated by extracellular sulfatases Sulf1 and Sulf2 in mice. *The Journal of Biological Chemistry*.

[4] Wang S., Ai X., Freeman S. D. (2004). QSulf1, a heparan sulfate 6-O-endosulfatase, inhibits fibroblast growth factor signaling in mesoderm induction and angiogenesis. *Proceedings of the National Acadamy of Sciences of the United States of America*.

[5] Ruhrberg C., Gerhardt H., Golding M. (2002). DT: Spatially restricted patterning cues provided by heparin-binding VEGF-A control blood vessel branching morphogenesis. *Genes & Development*.

[6] Chen E., Stringer S. E., Rusch M. A., Selleck S. B., Ekker S. C. (2005). A unique role for 6-O sulfation modification in zebrafish vascular development. *Developmental Biology*.

[7] Ashikari-Hada S., Habuchi H., Kariya Y., Kimata K. (2005). Heparin regulates vascular endothelial growth factor165-dependent mitogenic activity, tube formation, and its receptor phosphorylation of human endothelial cells. Comparison of the effects of heparin and modified heparins. *The Journal of Biological Chemistry*.

[8] Jakobsson L., Kreuger J., Holmborn K. (2006). Heparan sulfate in trans potentiates VEGFR-mediated angiogenesis. *Developmental Cell*.

[9] Gorsi B., Liu F., Ma X. (2014). The heparan sulfate editing enzyme Sulf1 plays a novel role in zebrafish VegfA mediated arterial venous identity. *Angiogenesis*.

[10] Gorsi B., Whelan S., Stringer S. E. (2010). Dynamic expression patterns of 6-O endosulfatases during zebrafish development suggest a subfunctionalisation event for sulf2. *Developmental Dynamics*.

[11] Eremina V., Sood M., Haigh J. (2003). Glomerular-specific alterations of VEGF-A expression lead to distinct congenital and acquired renal diseases. *The Journal of Clinical Investigation*.

[12] Eremina V., Cui S., Gerber H. (2006). Vascular endothelial growth factor A signaling in the podocyte-endothelial compartment is required for mesangial cell migration and survival. *Journal of the American Society of Nephrology*.

[13] Schumacher V. A., Schlötzer-Schrehardt U., Karumanchi S. A. (2011). WT1-dependent sulfatase expression maintains the normal glomerular filtration barrier. *Journal of the American Society of Nephrology*.

[14] Müller-Deile J., Schenk H., Niggemann P. (2019). Mutation of microphthalmia-associated transcription factor (mitf) in zebrafish sensitizes for glomerulopathy. *Biology Open*.

[15] Guan N., Ding J., Deng J., Zhang J., Yang J. (2004). Key molecular events in puromycin aminonucleoside nephrosis rats. *Pathology International*.

[16] Hanke N., Staggs L., Schroder P. (2013). “Zebrafishing” for novel genes relevant to the glomerular filtration barrier. *BioMed Research International*.

[17] Schenk H., Müller-Deile J., Kinast M., Schiffer M. (2017). Disease modeling in genetic kidney diseases: zebrafish. *Cell and Tissue Research*.

[18] Müller-Deile J., Schenk H., Schroder P. (2019). Circulating factors cause proteinuria in parabiotic zebrafish. *Kidney International*.

[19] Uchimura K., Morimoto-Tomita M., Bistrup A. (2006). HSulf-2, an extracellular endoglucosamine-6-sulfatase, selectively mobilizes heparin-bound growth factors and chemokines: effects on VEGF, FGF-1, and SDF-1. *BMC Biochemistry*.

[20] Eremina V., Jefferson J. A., Kowalewska J. (2008). VEGF inhibition and renal thrombotic microangiopathy. *The New England Journal of Medicine*.

[21] Zhou W., Hildebrandt F. (2012). Inducible podocyte injury and proteinuria in transgenic zebrafish. *Journal of the American Society of Nephrology*.

[22] Siegerist F., Blumenthal A., Zhou W., Endlich K., Endlich N. (2017). Acute podocyte injury is not a stimulus for podocytes to migrate along the glomerular basement membrane in zebrafish larvae. *Scientific Reports*.

