# Supplementary figures and images for: Sulfatases, in Particular Sulf1, Are Important for the Integrity of the Glomerular Filtration Barrier in Zebrafish
Source: Biomed Res Int. 2019 Jul 22;2019:4508048. doi: 10.1155/2019/4508048 (PMC6679890; doi:10.1155/2019/4508048)

## Slide 1
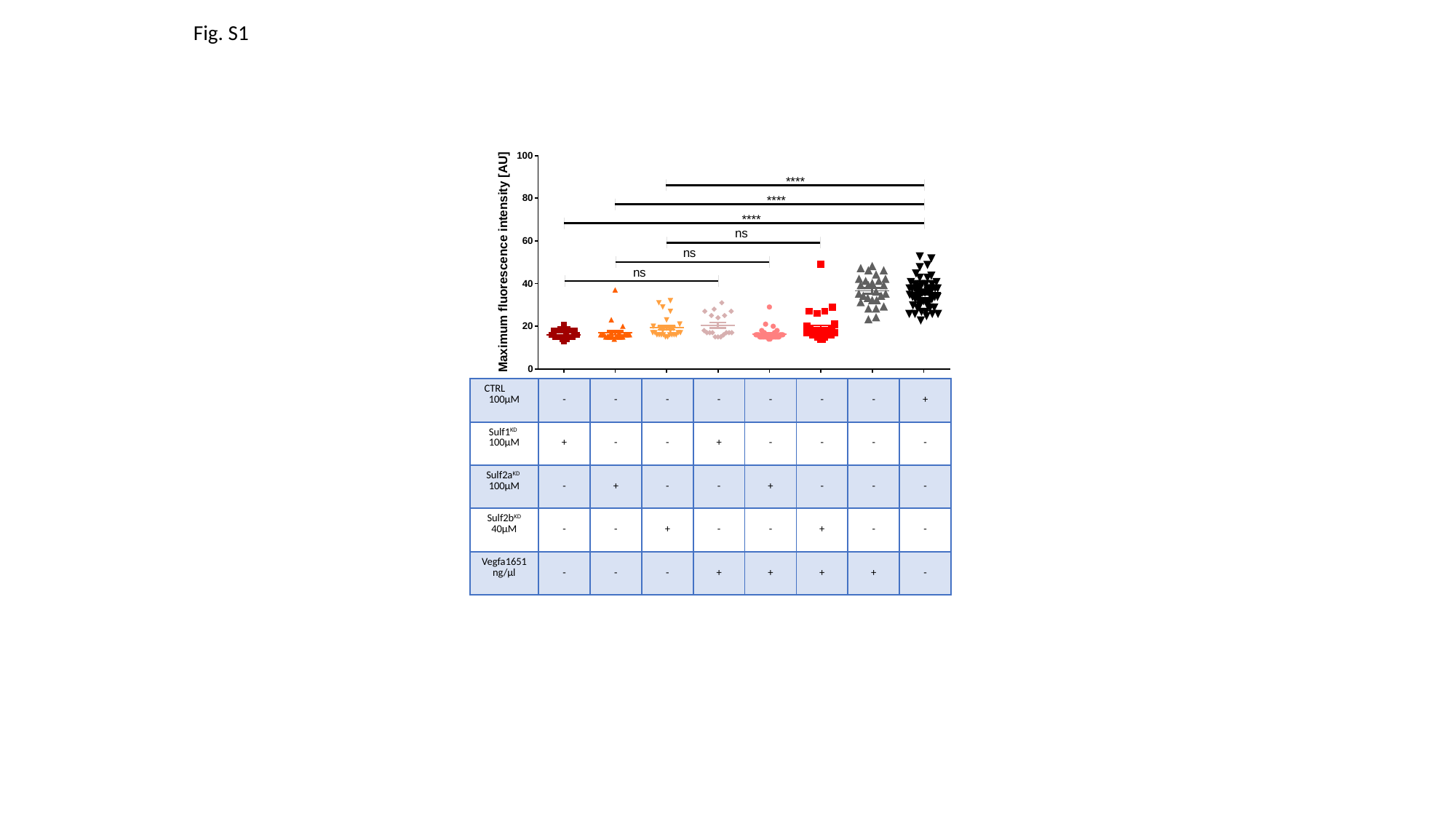

Fig. S1
| CTRL 100µM | - | - | - | - | - | - | - | + |
| --- | --- | --- | --- | --- | --- | --- | --- | --- |
| Sulf1KD 100µM | + | - | - | + | - | - | - | - |
| Sulf2aKD 100µM | - | + | - | - | + | - | - | - |
| Sulf2bKD 40µM | - | - | + | - | - | + | - | - |
| Vegfa1651 ng/µl | - | - | - | + | + | + | + | - |

Supplement: Supplementary Materials — Figure S1: Measurement of the maximum fluorescence intensity (AU) in the retinal vessel plexus at 96 hpf. Zebrafish embryos were injected in the yolk sac at the 1-2 cell stage with either a scrambled control (CTRL) or with sulf1 MO, sulf2a MO, or sulf2b MO and coinjected with Vegfa 165 protein. MO-induced Sulf1KD, Sulf2aKD, and Sulf2bKD induced maximum fluorescence intensity reduction indicative of eGFP-DBP loss; however, coinjection of Vegfa 165 1 ng/μl did not prevent fusion protein loss. Primers: Primer sequences for zebrafish: hprt forward: 5'ACCAAACACTATGCGGCTG3' and reverse: 5'GTGTCCACCCATGTCCTTCA3'. sulf1 forward: 5'AACCCACGATACAGACCTCG3' and reverse: 5'CCCCCTCAAACTCGACTGAC3' sulf2a forward: 5'ATTGTGAACCCACAGCGTCT3' and reverse: 5'TTCAGTTTCTTCTTTGTGAGTGCT3' sulf2b forward: 5'GACACCATGAGGTGCCAGAG3' and reverse: 5'TAAAACTGTGACCCGCGACG3'. Morpholinos: sulf1: GTAGTCCTGGTAGTGGTAGAATAAT sulf2a: CCATGACCTTAACCAGTGCTCCACC sulf2b: TTCAGTCTAATTCACCTTCTCCACA CTRL scrambled: CCTCCTACCTCAGTTACAATTTATA. [file 4508048.f1.pptx]
